# Gas Chromatography–Mass Spectrometry Profiling of Volatile Metabolites Produced by Some *Bacillus* spp. and Evaluation of Their Antibacterial and Antibiotic Activities

**DOI:** 10.3390/molecules28227556

**Published:** 2023-11-12

**Authors:** Moldir Koilybayeva, Zhanserik Shynykul, Gulbaram Ustenova, Krzysztof Waleron, Joanna Jońca, Kamilya Mustafina, Akerke Amirkhanova, Yekaterina Koloskova, Raushan Bayaliyeva, Tamila Akhayeva, Mereke Alimzhanova, Aknur Turgumbayeva, Gulden Kurmangaliyeva, Aigerim Kantureyeva, Dinara Batyrbayeva, Zhazira Alibayeva

**Affiliations:** 1 School of Pharmacy, S.D. Asfendiyarov Kazakh National Medical University, Tole-bi 94, Almaty 050012, Kazakhstan; g.ustenova@kaznmu.kz (G.U.); amirhanova.a@kaznmu.kz (A.A.); gulden-1207@mail.ru (G.K.); kantureeva.a@kaznmu.kz (A.K.); 2 Higher School of Medicine, Al-Farabi Kazakh National University, Almaty 050040, Kazakhstan; akhaeva.tamila@kaznu.kz (T.A.); turgumbayeva.aknur@med-kaznu.com (A.T.); 3 Department of Pharmaceutical Microbiology, Faculty of Pharmacy, Medical University of Gdańsk, Gen. Hallera 107, 80-416 Gdańsk, Poland; krzysztof.waleron@gumed.edu.pl (K.W.); joanna.jonca@gumed.edu.pl (J.J.); 4 Laboratory of Plant Protection and Biotechnology, Intercollegiate Faculty of Biotechnology University of Gdansk and Medical University of Gdańsk, University of Gdansk, 80-307 Gdańsk, Poland; 5 School of Medicine, S.D. Asfendiyarov Kazakh National Medical University, Tole-bi 94, Almaty 050012, Kazakhstan; mustafina.k@kaznmu.kz (K.M.); koloskova.e@kaznmu.kz (Y.K.); raukenty@mail.ru (R.B.); 6 Center of Physical Chemical Methods of Research and Analysis, Al-Farabi Kazakh National University, Almaty 050012, Kazakhstan; mereke.84@mail.ru; 7 Scientific Clinical Diagnostic Laboratory, S.D. Asfendiyarov Kazakh National Medical University, Tole-bi 94, Almaty 050012, Kazakhstan; dinarabat@mail.ru (D.B.); zhazira-alibaeva@mail.ru (Z.A.)

**Keywords:** antimicrobial activity, *Bacillus subtilis*, *Bacillus thuringiensis*, *Bacillus toyonensis*, *Bacillus acidiproducens*, *Bacillus cereus*, *Bacillus safensis*, GC–MC analysis, volatile organic compounds

## Abstract

*Bacillus* species produce different classes of antimicrobial and antioxidant substances: peptides or proteins with different structural compositions and molecular masses and a broad range of volatile organic compounds (VOCs), some of which may serve as biomarkers for microorganism identification. The aim of this study is the identification of biologically active compounds synthesized by five *Bacillus* species using gas chromatography coupled to mass spectrometry (GC–MS). The current study profoundly enhances the knowledge of antibacterial and antioxidant metabolites ensuring the unambiguous identification of VOCs produced by some *Bacillus* species, which were isolated from vegetable samples of potato, carrot, and tomato. Phylogenetic and biochemical studies were used to identify the bacterial isolates after culturing. Phylogenetic analysis proved that five bacterial isolates BSS12, BSS13, BSS16, BSS21, and BSS25 showed 99% nucleotide sequence similarities with *Bacillus safensis* AS-08, *Bacillus cereus* WAB2133, *Bacillus acidiproducens* NiuFun, *Bacillus toyonesis* FORT 102, and *Bacillus thuringiensis* F3, respectively. The crude extract was prepared from bacterial isolates to assess the antibiotic resistance potency and the antimicrobial potential against various targeted multidrug-resistant strains, including yeast strains such as *Candida albicans*, *Candida krusei*, and bacterial strains of *Enterococcus hirae*, *Escherichia coli*, *Klebsiella aerogenes*, *Klebsiella pneumoniae*, *Staphylococcus aureus*, *Staphylococcus epidermidis*, *Streptococcus* group B, *Streptococcus mutans*, *Shigella sonnei*, *Salmonella enteritidis*, *Serratia marcescens*, *Pseudomonas aeruginosa*, and *Proteus vulgaris*. GC–MS analysis of bacterial strains found that VOCs from *Bacillus* species come in a variety of chemical forms, such as ketones, alcohols, terpenoids, alkenes, etc. Overall, 69 volatile organic compounds were identified from five *Bacillus* species, and all five were found to share different chemical classes of volatile organic components, which have a variety of pharmacological applications. However, eight antibacterial compounds with different concentrations were commonly found in all five species: acetoin, acetic acid, butanoic acid, 2-methyl-, oxime-, methoxy-phenyl, phenol, 1,2-benzenedicarboxylic acid, bis(2-methylpropyl) ester, nonanoic acid, and hexadecanoic acid, methyl. The present study has demonstrated that bacterial isolates BSS25, BSS21, and BSS16 display potent inhibitory effects against *Candida albicans*, while BSS25, BSS21, and BSS13 exhibit the ability to restrain the growth and activity of *Candida krusei*. Notably, BSS25 and BSS21 are the only isolates that demonstrate substantial inhibitory activity against *Klebsiella aerogenes*. This disparity in inhibitory effects could be attributed to the higher concentrations of acetoin in BSS25 and BSS21, whereas BSS16 and BSS13 have relatively elevated levels of butanoic acid, 2-methyl-. Certainly, the presence of acetoin and butanoic acid, 2-methyl-, contributes to the enhanced antibacterial potential of these bacterial strains, in conjunction with other organic volatile compounds and peptides, among other factors. The biology and physiology of *Bacillus* can be better understood using these results, which can also be used to create novel biotechnological procedures and applications. Moreover, because of its exceptional ability to synthesize and produce a variety of different antibacterial compounds, *Bacillus* species can serve as natural and universal carriers for antibiotic compounds in the form of probiotic cultures and strains to fight different pathogens, including mycobacteria.

## 1. Introduction

The emergence of bacterial strains that previously were susceptible to existing antibiotics but now cause serious infectious diseases makes it necessary to find and create novel treatments for these illnesses [1]. The most well-known and clinically significant example of this issue is the rise in multidrug-resistant strains of *Staphylococcus aureus* (MRSA), which is most pathogenic and leads to the formation of an abscess. Moreover, it can cause pneumonia, endocarditis, and osteomyelitis. According to some investigations, MRSA is resistant not only to some antibiotics such as methicillin, macrolides, tetracycline, aminoglycosides, and chloramphenicol, but also to some disinfectants [2].

In order to create new antibiotic treatments or disinfectants, it is also necessary to look for and analyze compounds that have bactericidal or bacteriostatic capabilities against human and animal infections. Currently, analysis of the potential of natural compounds from various sources as antimicrobials has received significant attention in addition to the synthesis of new chemical substances. The observation of antagonism, or the interaction between microorganisms, is frequently the starting point for the development of antibiotics with activity against human infections. The creation and release of chemicals that impede or entirely stop the growth of other species serve as the physical manifestation of this hostility. Under natural circumstances, an agent released by a microbe that prevents the growth of another organism has an edge in the competition for environmental resources. The majority of antibiotics used in medicine are secreted by or derived from bacteria. Hence, it is a fact that the bacterial world has a vast repository of potentially antimicrobial chemicals that have not yet been identified or exploited. In this respect, members of the genus *Bacillus* are recognized as manufacturers of a wide variety of enzymes and antibacterial substances. For example, 23 peptide antibiotics are produced by *Bacillus brevis*, while *Bacillus subtilis* produces roughly 70 antimicrobials, which are ribosomal peptides, non-ribosomal peptides, polyketides, hybrids, and volatile compounds [3,4]. Consequently, there is increased interest in taking these compounds into consideration as disjunctive antimicrobials for the healing of human infections [5,6,7,8,9].

Nowadays, a novel strategy for the management and prevention of numerous infectious illnesses is the use of bacterial probiotic strains and their metabolic products [10]. Probiotics from the *Bacillus* genus have been shown to exhibit antibacterial effects in experiments on animals [11,12]. As an affordable and infrequently resistant alternative to antibiotics, the application of bacteriocins and antimicrobial peptides synthesized by probiotic strains is recommended [13,14]. They hold promise for clinical usage since many of these molecules are efficient and affordable [15]. Due to their desirable medicinal qualities, such as their antibacterial, antiviral, anticancer, and contraceptive effects, a few natural peptides from bacterial isolates have demonstrated potential. Furthermore, when combined with traditional antibiotics, they have been demonstrated to offer protection against systemic and topical infections. Therefore, the justification for using probiotics in medicine is founded on the notion that administering oral or topically applied probiotics could restore the depleted state of the human microbiome [16].

The development, registration, and commercialization of biocontrol drugs based on microbial antagonists have advanced significantly during the past few decades. Although their use has side effects for both human and animal health. *Bacillus* species compete directly with fungal pathogens for resources and habitats and through a variety of processes such as the generation of siderophores. Hence, they also indirectly create systemic resistance or stimulate the growth of plants [17,18]. Moreover, they produce a vast array of volatile organic compounds with strong inhibitory potential against plant pathogens: alcohols, alkenes, benzenoids, terpenoids, ketones, sulfur-containing compounds, and others [19,20]. The non-volatile components of these metabolites have received a great deal of scientific interest, whereas the volatile components are examined less frequently. Numerous applications in biology, environmental sciences, health, the food industry, and national security include the study and detection of volatile organic compounds (VOCs) that come from or interact with creatures ranging from bacteria to people. Low-molecular-weight organic molecules with a lipophilic nature and a low boiling point are known as volatile organic compounds (VOCs) [21]. According to several studies, VOCs released by bacteria may help plants by fostering development, triggering defense mechanisms, and inhibiting or removing dangerous infections [22,23,24,25,26]. Furthermore, VOCs released by microorganisms are biodegradable as they are naturally occurring compounds. As a result, using VOCs produced by microorganisms is a sustainable method of crop protection and promotion. According to some recent studies, because they can stop certain pathogenic fungi’s mycelial growth and spore germination, VOCs produced by *B. subtilis* have been suggested as an alternate control approach for postharvest fruit illnesses [27]. For instance, different VOCs produced by *B. subtilis* TB09 and TB72, such as nonan-2-one, β-benzeneethanamine, and 2-methyl-1,4-diazine effectively controlled the anthracnose pathogen on postharvest mangoes [28]. Likewise, some VOCs synthesized by *B. subtilis* PPCB001 have helped to evaluate its antagonistic activity and it was found that *B. subtilis* PPCB001 reduces the growth of one of the imperfect fungi, which is called *Penicillium crustosum* [29]. Additionally, the GC–MS analysis of three bacterial isolates of *Bacillus subtilis* Md1-42, *Bacillus subtilis* O-3, and *Bacillus subtilis* Khozestan2 samples proved the presence of phenol, benzoic acid, 1,2-benzenedicarboxylic acid, bis(2-methylpropyl), methoxyphenyl-oxime, and benzaldehyde, which are known for their antimicrobial and other properties [30].

Overall, representatives of the genus *Bacillus* have been found to be producers of a wide range of antimicrobial compounds. The synergetic mechanism of antimicrobial compounds such as VOCs, polyketides, ribosomal peptides, and others explains why they have an increased industrial interest as therapeutic agents, food preservatives and biopesticides. Since *Bacillus* species have the unique ability to produce a variety of diverse antibacterial chemicals, they can serve as a natural carrier for antibiotics in the form of probiotic cultures and strains to fight different pathogens, including mycobacteria. Nevertheless, the dangerous effects of several antibacterials on humans and animals have prevented some of them from being used medically despite their promising in vitro antimycobacterial activity [31,32].

Another instance of *Bacillus* species employment is in the manufacture of food-grade amylase, glucoamylase, protease, pectinase, and cellulase for a variety of foods [33,34,35,36]. Additionally, many species of *Bacillus* have been employed to synthesize a number of dietary supplements for human use, including vitamins (such as riboflavin, cobalamin, and inositol) and carotenoids [36,37,38,39]. However, despite these advantages, these strains have not attracted much interest in the contemporary functional food market because of their relationships with a limited number of human diseases.

This study focused on isolating potential *Bacillus* species from vegetable samples (potato, carrot, and tomato) and preparing a crude extract from isolated bacterial strains to assess antimicrobial activities against the most common human pathogens. In addition, bacterial isolates were tested for antibiotic resistance using an inhibition zone diameter when determined via the disk diffusion method. GC–MC analysis was performed to determine bioactive compounds from the bacterial isolates. This study will facilitate the development of novel antibiotics against MDR bacterial strains and help to explore possible probiotics typical for the representatives of the genus *Bacillus*.

The novelty of this work lies in several aspects. Firstly, it highlights the potential of these isolated *Bacillus* strains to exhibit antibacterial activity, particularly against multidrug-resistant bacterial strains. This is crucial in the context of the growing problem of antibiotic resistance, as these strains can serve as valuable resources for the development of new antimicrobial agents. Secondly, the study identifies specific bioactive compounds present in the bacterial extracts using GC–MC analysis. These compounds have diverse antifungal, antioxidant, and anticancer effects. This provides a basis for understanding the potential therapeutic applications of these compounds in various fields, including medicine and agriculture. Additionally, the research emphasizes the presence of certain substances, such as acetic acid, benzaldehyde and acetoin, as intracellular compounds within bacterial cells, in addition to their role as volatile substances. Understanding the intracellular presence and functions of these compounds contributes to the broader knowledge of bacterial metabolism and the potential utilization of these compounds in various applications.

In summary, the study combines microbial screening, compound identification, and antibacterial activity assessment to offer insights into the taxonomy, antimicrobial potential, and chemical composition of these *Bacillus* strains. The findings increase the importance and significance of the work and hold promise for addressing challenges related to infectious diseases, antibiotic resistance, and the development of novel therapeutic agents. This research represents a valuable contribution to the field of microbiology and biotechnology.

## 2. Results

### 2.1. Isolation and Identification

A total of *n* = 25 bacteria strains were isolated and identified using colony morphology, microscopy, biochemical properties, and sugar fermentation. From among these, Gram-strain-positive, rod-shaped, mycelial, and spore-forming bacterial strains were chosen for further verifying tests. The molecular analysis further validated the bacterial strains (BSS25, BSS21, BSS16, BSS13, and BSS12) as *Bacillus thuringiensis* F3, *Bacillus toyonensis* FORT 102, *Bacillus acidiproducens* NiuFun, *Bacillus cereus* WAB2133, and *Bacillus safensis* AS-08.

### 2.2. Microbial Morphology and Colony Characteristics

The morphology of each colony by different bacterial isolates showed regular, irregular, slightly raised, flat, white, and cream-colored colonies. By motility test, bacterial isolates were motile and possessed terminal and subterminal spores (Table 1).

### 2.3. Antimicrobial Potency Evaluation

**Figure 1:**
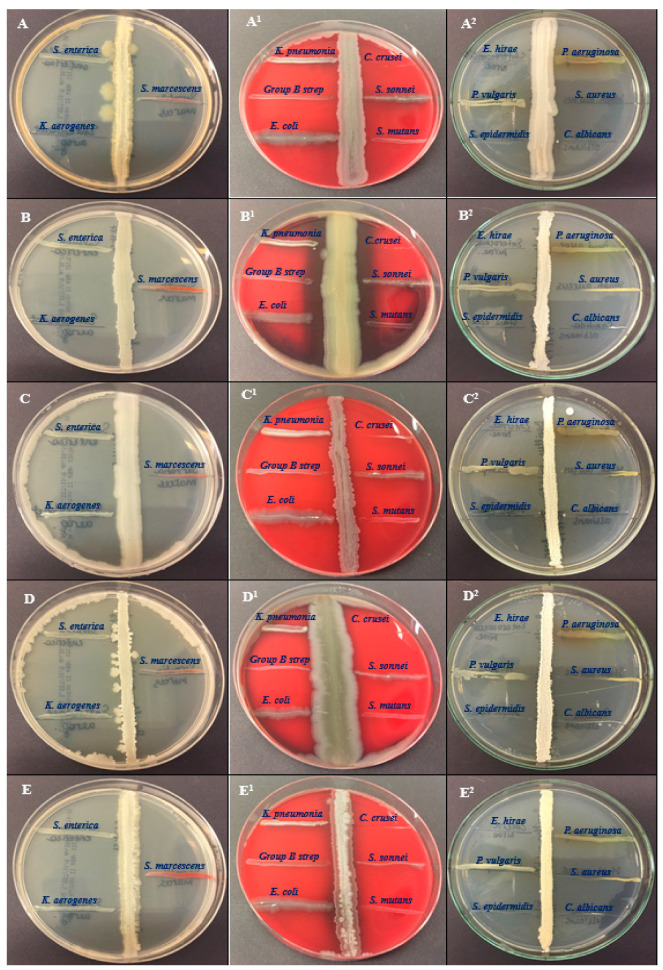
Antagonistic activity of the bacteria of the genus *Bacillus* against pathogens. The antagonistic efficacy of all five isolates was examined against pathogenic bacteria, such as *Salmonella enterica* ATCC 35664, *Serratia marcescens* ATCC 13880, *Klebsiella aerogenes* ATCC 13048, *Shigella sonnei* ATCC 25931, *Streptococcus mutans* ATCC 25175, *Klebseiella pneumoniae* ATCC 13883, Group B *Streptococcus*, *Escherichia coli* ATCC 25922, *Pseudomonas aeruginosa* ATCC 9027, *Staphylococcus aureus* ATCC 29213, *Enterococcus hirae* ATCC 10541, *Proteus vulgaris* ATCC 6380, and *Staphylococcus epidermidis* ATCC 12228. Moreover, the antagonistic efficacy of all five isolates was examined against yeast strains such as *Candida albicans* ATCC 2091 and *Candida krusei* ATCC 14243. (**A**–**A2**)—BSS25, (**B**–**B2**)—BSS21, (**C**–**C2**)—BSS16, (**D**–**D2**)—BSS13, and (**E**–**E2**)—BSS12.

Five bacterial cultures of BSS25, BSS21, BSS16, BSS13 and BSS12 were tested for their antagonistic activity against 15 pathogens such as *Salmonella enterica* ATCC 35664, *Klebsiella aerogenes* ATCC 13048, *Serratia marcescens* ATCC 13880, *Klebsiella pneumoniae* ATCC 13883, *Streptococcus group B, Escherichia coli* ATCC 25922, *Candida krusei* ATCC 14243, *Shigella sonnei* ATCC 25931, *Streptococcus mutans* ATCC 25175, *Enterococcus hirae* ATCC 10541, *Proteus vulgaris* ATCC 6380, *Staphylococcus epidermidis* ATCC 12228, *Pseudomonas aeruginosa* ATCC 9027, *Staphylococcus aureus* ATCC 29213, and *Candida albicans* ATCC 2091 (Figure 1).

The five extracts showed antibacterial activity against all the bacterial pathogens except *Staphylococcus epidermidis* ATCC 12228 and *Enterococcus hirae* ATCC 10541 (Table 2). The strain of *Bacillus thuringiensis* F3 (BSS25), showed a better zone of inhibition for *Staphylococcus aureus* ATCC 29213 (35 ± 1.27 mm), *Staphylococcus epidermidis* ATCC 12228 (37 ± 1.47 mm), *Candida albicans* ATCC 2091 (36 ± 1.43 mm), *Candida krusei* ATCC 14243 (37 ± 1.41 mm), *Klebsiella aerogenes* ATCC 13048 (37 ± 1.27 mm), and *Enterococcus hirae* ATCC 10541 (37 ± 1.25 mm). Additionally, the strain of *Bacillus toyonensis FORT 102* (BSS21) was effective against the pathogens *Staphylococcus epidermidis* ATCC 12228 (36 ± 1.27), *Candida albicans* ATCC 2091 (38 ± 1.21 mm), *Candida krusei* ATCC 14243 (36 ± 1.28 mm), *Klebsiella aerogenes* ATCC 13048 (36 ± 1.37 mm), and *Enterococcus hirae* ATCC 10541 (38 ± 1.27 mm).

### 2.4. Antibiotic Susceptibility Profile of the Isolates

With the exception of bacitromycin (B, 10), polymyxin (PB, 300), and cloxacillin (CX, 5), none of the five *Bacillus* species examined were resistant to any antibiotics, according to the analysis of the antibiogram (Table 3). All five strains, which are BSS25, BSS21, BSS16, BSS13, and BSS13, showed the highest vulnerability to gentamicin (CN, 120) with 39 ± 0.37 mm, 38 ± 0.43 mm, 40 ± 0.12 mm, 41 ± 0.23, and 39 ± 0.31 sensitivity diameters, respectively.

### 2.5. GC–MS Analysis

According to the results of the GC–MS analysis, crude extracts from different *Bacillus* bacterium species contained a variety of compounds. Table 4, Table 5, Table 6, Table 7 and Table 8 explain the most important and plentiful components identified in the crude extracts that were subjected to the GC–MS analysis, as well as information about where the chemicals found in this study had previously been identified. These substances exhibited similarities to the natural products of a variety of organisms, such as of bacterial, plant and fungi origin. In one study, the majority of the compounds detected were derived from volatile substances. Different *Bacillus* species have been found to produce volatile compounds belonging to various classes, such as alcohols, ketones, fatty acids, and aromatic compounds, in addition to esters and ethers. In strain BSS25, ethyl acetate extraction showed the presence of 33 compounds (Table 4) compared to 37 compounds arising out of the same extraction strain BSS21 (Table 5). In the ethyl acetate extract of the BSS16 bacterial strain, 23 compounds were identified (Table 6). Acetoin, benzaldehyde, 3(2H)-thiophenone, dihydro-2-methyl-, propanoic acid, 2-methyl-, and oleic acid were identified in the BSS16 extract with important concentrations of 8.44%, 4.75%, 6.07%, 13.98%, and 9.68%, respectively. In the BSS25 bacterial isolate, the major compounds were butanoic acid, 2-methyl- at 29.39% and 9,12-octadecadienoic acid (Z, Z)- at 11.10% and three other compounds such as 3(2H)-thiophenone, dihydro-2-methyl-, benzoic acid, tridecyl ester, and pentadecanoic acid were identified only in this bacterial extract. In bacterial strain BSS13, some compounds such as acetone, acetic acid, benzaldehyde, hexadecanoic acid, octadecanoic acid, 2-hydroxy-1,3-propanediyl ester, 9-Octadecenoic acid, (E)-, and 9,12-Octadecadienoic acid (Z, Z)- were found with high concentrations 3.66%, 6.31%, 6.24%, 4.45%, 3.79%, 9.95%, and 5.87%, respectively (Table 7). The solvent with metabolites for isolate BSS12 was ethyl acetate, which also contained 38 chemicals (Table 8), whereas the same solvent with metabolites for isolate BSS13 was found to contain 33 compounds (Table 7). GC–MS analysis for five bacterial (BSS25, BSS21, BSS16, BSS13 and BSS12) analyses also confirmed the presence of the same volatile organic compounds, while some components were found only in some bacterial isolates.

The volatile organic compounds (VOCs) found in high concentrations in *Bacillus* species are a diverse array of chemical compounds that contribute to the distinct odor and metabolic functions of these bacteria. These VOCs vary between *Bacillus* species, influenced by genetic factors, environmental conditions, and growth stages. Common VOCs produced by *Bacillus* species include alcohols, aldehydes, and esters, which can have applications in food preservation, antimicrobial activities, and even as biocontrol agents in agriculture. Furthermore, certain *Bacillus* strains are recognized for their unique VOC profiles, making them valuable for biotechnological and industrial processes, as well as in fields such as bioremediation, where VOCs can aid in the breakdown of pollutants. The specific compounds found in high concentrations in all five bacterial extracts are shown in Table 9.

The purity of the extract obtained from all five Bacillus species was assessed by GC-MS analysis. The examination unveiled prominent peaks that corresponded to various other compounds, with purities varying between 60% and 95%. For instance, the analysis revealed a major peak corresponding to acetoin for *Bacillus thuringiensis* (BSS25), *Bacillus toyonensis* (BSS21), *Bacillus acidiproducens* (BSS16), *Bacillus cereus* (BSS13), and *Bacillus safensis* with a purity of 89% as determined by peak area integration. No significant impurities exceeding the threshold were detected. Overall, the level of purity ensures the reliability of the GC-MS results for the identification and quantification of target compounds.

**Figure 2:**
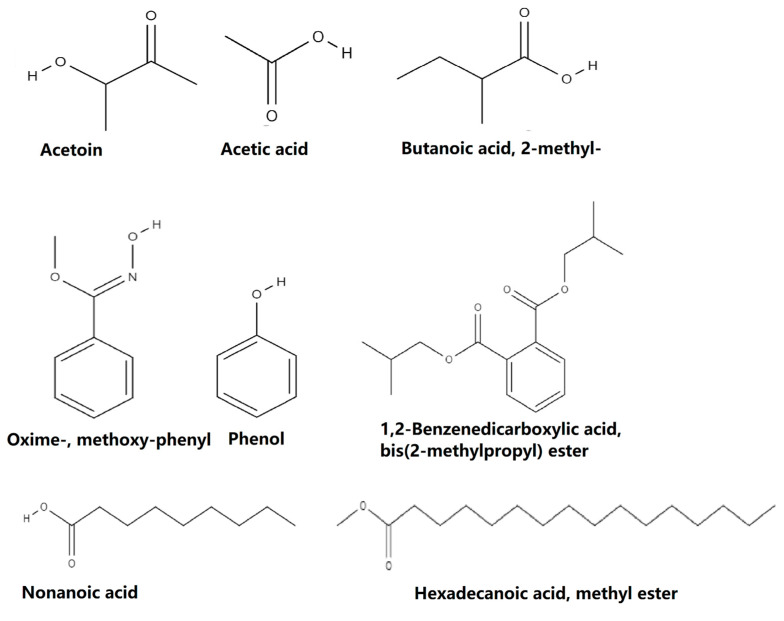
Structure of common components identified from *Bacillus* spp. isolates.

The GC–MS based metabolite profiling of the ethyl acetate extracts of BSS25, BSS21, BSS16, BSS13, and BSS12 bacterial isolates revealed a total of 69 volatile organic substances (Table 10). Based on the analysis of bacterial isolates, all five isolates were found to share a similar composition of volatile organic components, such as acetoin, acetic acid, butanoic acid, 2-methyl-, oxime-, methoxy-phenyl, phenol, 1,2-benzenedicarboxylic acid, bis(2-methylpropyl) ester, nonanoic acid, and hexadecanoic acid, methyl ester. Their chemical structure details are available, as illustrated in Figure 2.

*Bacillus* is a diverse genus of bacteria, and different species and strains within this genus can exhibit varying metabolic capabilities, including the production of volatile organic compounds (VOCs) and antimicrobial compounds. Taxonomy, especially at the species and strain level, can influence the types and quantities of volatile substances produced by *Bacillus* strains. Different species of *Bacillus* may have distinct metabolic pathways and genetic backgrounds that lead to the production of specific VOCs. The antimicrobial activity of *Bacillus* strains is often linked to the production of secondary metabolites, including antibiotics and antimicrobial peptides. The taxonomic classification of *Bacillus* strains can provide insights into their potential to produce these compounds. For instance, it was found that the bacterial extract of *Bacillus thuringiensis* composes such antimicrobial compounds of hexanal, 1-hepten-4-ol, and 1-decanol. Moreover, compounds of 5,9-undecadien-2-one, 6,10-dimethyl-, (E)-,propanoic acid, 2-methyl-, 3-hydroxy-2,4,4-trimethylpentyl ester, and cetene were found only in the bacterial extract of *Bacillus thuringiensis*, but their pharmacological activities have not been studied, yet. Antibacterial compounds such as nonanal, pentadecanoic acid, 1-dodecanol, 3-buten-2-one, 4-(1-cyclopenten-1-yl)-, (E)-, 2,3-pentanedione, E-3-pentadecen-2-ol, 2-octanol, and dodecanal were found only in the *Bacillus toyonensis* extract. Among these compounds, nonanal, pentadecanoic acid and 1-dodecanol are known for their antifungal and antimicrobial properties and the pharmacological activity of the remaining compounds remains unexplored due to a lack of available information. *Bacillus acidiproducens* extract differs from the other five bacterial extracts due to the presence of 3(2H) thiophenone, dihydro-2-methyl- and pentadecanoic acid. Pentadecanoic acid is a JAK2/STAT3 signaling inhibitor in breast cancer cells [59] and an anti-biofilm agent [60]. However, the antimicrobial and other pharmacological properties of 3(2H) thiophenone, dihydro-2-methyl, have not been previously documented or reported. Based on our research, 9-Octadecenoic acid, (E)-, and octadecanoic acid, 2-hydroxy-1,3-propanediyl ester were uniquely identified in *Bacillus cereus* and were not found in *Bacillus thuringiensis*, *Bacillus toyonensis*, *Bacillus acidiproducens,* or *Bacillus safensis.* Lastly, the extract from *Bacillus safensis* stands out as unique compared to extracts from the other bacteria due to the presence of distinctive compounds, including (2-aziridinylethyl)amine, 1-propen-2-ol, acetate, 3-penten-1-ol, 2-nonen-1-ol, 2-hydroxy-3-pentanone, ethane-1,1-diol dibutanoate, 2,3-butanediol, and benzoic acid, undecyl ester.

Volatile substances can be used as antimicrobials through various mechanisms and applications, depending on their specific properties. VOCs with antimicrobial properties can be used to inhibit the growth of microorganisms when they come into contact with them. For example, these compounds can be incorporated into antimicrobial surfaces, such as coatings, films, or textiles, to prevent the growth of bacteria or fungi on these surfaces. Additionally, VOCs can be employed for fumigation in enclosed spaces. For instance, essential oils containing volatile antimicrobial compounds can be vaporized to disinfect indoor environments, like hospital rooms or food storage areas. This approach can help reduce the microbial load in the air and on surfaces. Moreover, VOCs can be used as active ingredients in pharmaceuticals and personal care products, such as antimicrobial creams, lotions, and inhalers. It is important to note that the effectiveness of volatile substances such as antimicrobials can vary depending on factors such as the specific compound, concentration, targeted microorganisms, and environmental conditions.

### 2.6. Molecular Characterization

**Figure 3:**
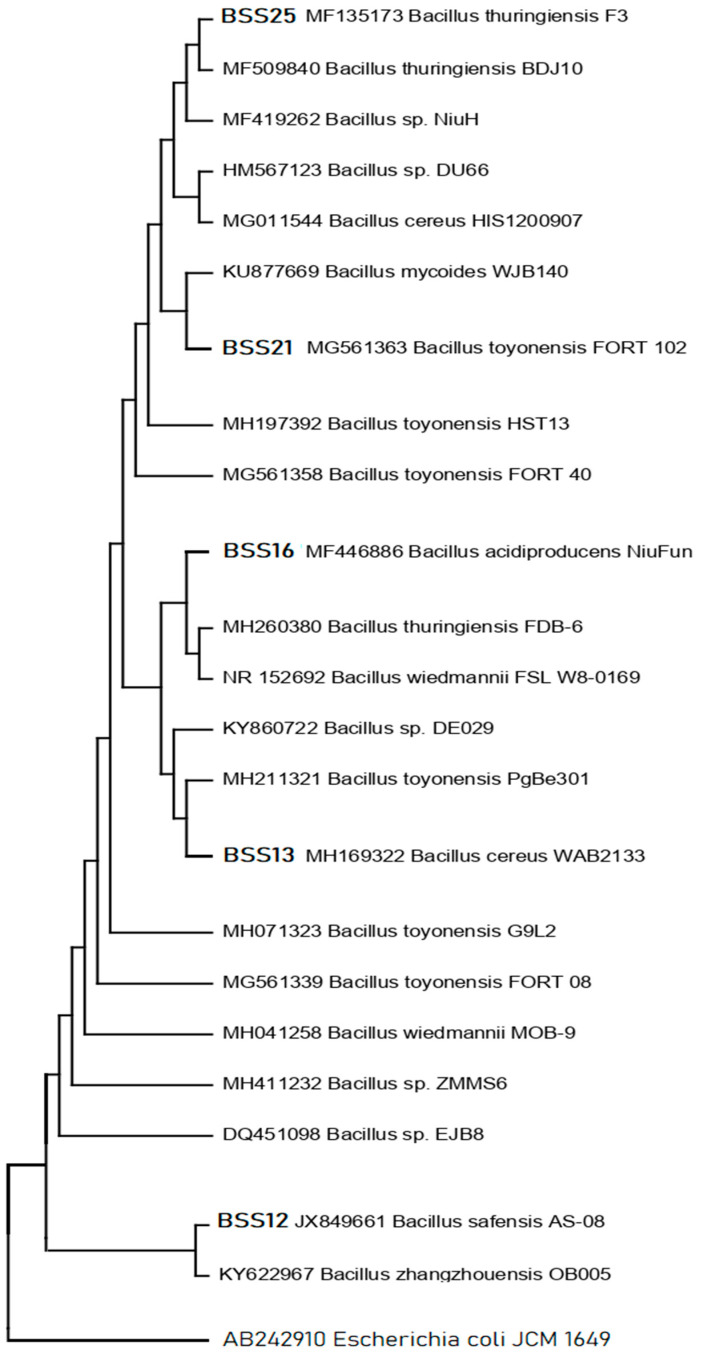
The phylogenetic tree using the neighbor-joining model was constructed according to 16S rRNA gene sequences representing different *Bacillus* species, i.e., *Bacillus thuringiensis* F3, *Bacillus toyonensis* FORT 102, *Bacillus acidiproducens* NiuFun, *Bacillus cereus* WAB2133, and *Bacillus safensis* AS-08, respectively. *E. coli* JCM 1649 (AB242910) was used as an outgroup in the phylogenetic tree.

Five (*n* = 5) bacterial isolates with increased antibacterial activity were isolated from distinct samples. Phylogenetic analysis of the 16S rRNA gene sequences indicated that all five candidate bacterial isolates, BSS25, BSS21, BSS16, BSS13, and BSS12, belong to five different *Bacillus* spp., respectively (Figure 3), as they are related to the aforementioned bacterial species in the phylogenetic tree.

The *Bacillus* species *Bacillus thuringiensis* F3, *Bacillus toyonensis* FORT 102, *Bacillus acidiproducens* NiuFun, *Bacillus cereus* WAB2133, and *Bacillus safensis* AS-08 were identified as having the highest hit sequence similarity for these bacterial isolates (Table 11). High bootstrap values were obtained following a phylogenetic analysis and tree topology and both served to confirm the described taxonomy.

## 3. Discussion

Microorganisms, including bacteria, archaea, fungi, and even viruses, inhabit diverse environments and contribute to the cycling of nutrients and the production of a wide array of metabolites. In particular, extreme microbial diversity, abundance, and structure can have significant implications for the production of various metabolites with diverse functions, including anti-parasitic, antimicrobial, pesticidal, and anti-cancerous functions. Consequently, these metabolites can have important applications in various fields. The goal of the present study was to investigate the possibility of particular vegetable microbial communities displaying antibacterial properties and to establish the possible relationship between isolated compounds through GC–MS and the antagonistic activity of the studied bacterial strains. As a result of various phases of isolation, the identification of diverse general objectives with the selection of bacterial growth conditions, and biochemical tests, nineteen (*n* = 19) different bacterial isolates were detected. In recent years, the ongoing exploration of microbial diversity, along with advancements in culturing techniques, genomics, and metagenomics, has rejuvenated the search for new antibiotics. This is a promising development in the fight against infectious diseases and antibiotic resistance, as it offers the potential for a new generation of antimicrobial agents to tackle previously untreatable infections [75,76]. However, both mobile genetic elements and inherent characteristics (natural phenotypic traits) contribute to the development and spread of antibiotic resistance, making it a complex and evolving problem in healthcare and public health. The inappropriate use of antibiotics, both in clinical settings and in agriculture, accelerates the selection and dissemination of antibiotic-resistant bacteria by providing a selective advantage to those carrying resistance genes. Addressing antibiotic resistance requires a multifaceted approach that includes responsible antibiotic use, surveillance, development of new antibiotics, and strategies to prevent the spread of resistant bacteria [77]. Nearly all antibiotics, with the exception of bacitracin, polymyxin, and cloxacillin, were found to be effective against *Bacillus species*, i.e., *Bacillus thuringiensis* (BSS25), *Bacillus toyonensis* (BSS21), *Bacillus acidiproducens* (BSS16), *Bacillus cereus* (BSS13), and *Bacillus safensis* (BSS12) (Table 3). Similar findings on the susceptibility of various antibiotic-susceptible *Bacillus* species were observed in some recent studies [30,78,79,80,81]. Here, resistance in specific *Bacillus* strains to particular antibiotics can result from both inherent (natural) mechanisms and acquired resistance due to the presence of resistance genes associated with the production of resistance enzymes [82]. The probability of passing on resistance genes to other bacteria, particularly dangerous pathogens, may be lower when resistance is due to inherent (natural) resistance mechanisms rather than acquired resistance through the acquisition of resistance genes. This distinction is important in the context of antibiotic resistance transmission and the potential for the development of multidrug-resistant or extensively drug-resistant bacteria. Antibiotic resistance has indeed become a serious global concern, and the spread of resistant bacteria through the food chain is one of the pathways contributing to this problem [83]. Although, isolated *Bacillus* strains may not necessarily harbor antibiotic resistance genes that can be horizontally transferred to dangerous pathogens, they can still exhibit natural resistance or insensitivity to a wide variety of antibiotics due to their inherent characteristics. Indeed, further research into *Bacillus* strains, especially those with unique characteristics or inherent resistance to antibiotics, can be valuable for various applications, including the development of probiotic starter cultures and the production of high-quality, medicinal, and health-promoting substances.

GC–MS is a powerful analytical technique commonly used to detect and identify various compounds in biological samples, including microbial cells and their metabolites helped in this study to detect markers in biological material, such as components of microbial cells and metabolites like fatty acids, aldehydes, and phenolic compounds. Additionally, using GC–MS without the need for the preliminary isolation of pure cultures of microorganisms offers several advantages in the case of both endogenous and exogenous microflora, which is especially important when considering the difficulties in cultivating anaerobes. The method’s unique benefits were quick analytical times and the capacity to quantify marker content. According to the GC–MS analysis, the *Bacillus* species produce a variety of chemicals (Table 9), which possess different pharmacological activities such as antiviral, antibacterial, antifungal, antioxidant, anticancer, anti-inflammatory, hyperlipidemic, antimicrobial, antinociceptive, analgesic, anxiolytic, antidepressive, neuroprotective, and so forth. Overall, 69 compounds were determined by the GC–MS analysis of their crude metabolites from five *Bacillus* species. Eight biologically active compounds such as acetoin, acetic acid, butanoic acid, 2-methyl-, oxime-, methoxy-phenyl, phenol, 1,2-benzenedicarboxylic acid, bis(2-methylpropyl) ester, nonanoic acid, and hexadecanoic acid, methyl ester were found to be common to all five strains (Table 4, Table 5, Table 6, Table 7 and Table 8 and Figure 2).

Previously, we found that acetic acid is present in bacterial isolates such as *Bacillus subtilis* O-3, *Bacillus subtilis* Md1-42, and *Bacillus subtilis* Khozestan2 [30]. According to the present study, acetic acid seems to be a common organic compound in almost for all *Bacillus* species as its presence has been confirmed in the other five *Bacillus* species, i.e., *Bacillus toyonensis*, *Bacillus acidiproducens*, *Bacillus cereus*, and *Bacillus safensis*. BSS13 has the highest concentration of acetic acid at 6.31%, while the other four strains were found to share similar low concentrations. Acetic acid is a common organic acid and a component of the volatile organic compounds (VOCs) produced by some bacterial species, including certain strains of *Bacillus*. Acetic acid is a byproduct of microbial metabolism, particularly in bacteria that undergo fermentation processes or produce acetic acid as part of their metabolic pathways. It has been known for its antibacterial and antifungal, anticancer activities [50]. The second compound common to all five strains was acetoin, and its concentrations for BSS25, BSS21, BSS16, BSS13, and BSS12 were as follows: 44.06%, 38.25%, 8.44%, 0.75%, and 0.26%, respectively. Acetoin is a common compound produced by various bacteria, and its concentration can vary among different strains. It is not typically used as a central nervous system (CNS) depressant in medical practice or for recreational purposes; however, in one recent study, it was proven that acetoin has a potent CNS depressant effect [44]. It was found that the third component that all bacteria share is butanoic acid, 2-methyl-, which has an application as a laxative. BSS16 and BSS13 have the highest concentrations of butanoic acid, 2-methyl- at 29.39% and 31.69%, while BSS16, BSS13, and BSS12 share very low concentrations at 2.24%, 3.57%, and 1.89%, respectively. The other four compounds common to all five strains, which are oxime-, meth-oxy-phenyl, 1,2-benzenedicarboxylic acid, bis(2-methylpropyl) ester, nonanoic acid, and hexadecanoic acid, methyl ester, were found at similarly low concentrations.

The GC–MS analysis of *Bacillus thuringiensis* has revealed the presence of several chemical compounds of 2,3-butanedione, (R, R)-2,3-butanediol, 1-hepten-4-ol, hexanoic acid, hexanoic acid, 2-ethyl-, octanoic acid, benzaldehyde, and (S)-(+)-6-methyl-1-octanol in relatively high concentrations at 15.85%, 4.08%, 3.42%, 1.86%, 2.90%, 2.47%, 2.22%, and 1.25%, respectively. The compositional study of *Bacillus toyonensis* established that it differs from other bacterial strains by containing 2,3-butanedione (16.97%), propanoic acid, 2-methyl- (1.41%), hexadecanoic acid (4.00%), oleic acid (5.85%), 9,12-octadecadienoic acid (Z, Z)- (3.80%), 2,3-pentanedione (2,04%), 3-pentanol, 2-methyl- (2.92%), oxirane, (methoxymethyl)- (2.41%), (R, R)-2,3-butanediol (7.80%), and hexadecanoic acid (4.00%). The presence of some valuable compounds in *Bacillus acidiproducens* also was confirmed, these being benzaldehyde (4.75%); 3(2H)-thiophenone, dihydro-2-methyl- (6.07%), propanoic acid, 2-methyl- (13.98%), hexadecanoic acid (3.61%), octadecanoic acid (1.64%), oleic acid (9.68%), and 9,12-octadecadienoic acid (Z, Z)- (11.10%). The presence of some valuable compounds in *Bacillus acidiproducens* also was confirmed, these being benzaldehyde (4.75%); 3(2H)-thiophenone, dihydro-2-methyl- (6.07%), propanoic acid, 2-methyl- (13.98%), hexadecanoic acid (3.60%), octadecanoic acid (1.64%), oleic acid (9.68%), and 9,12-octadecadienoic acid (Z, Z)- (11.10%), and the presence of these chemical constituents indicates the diverse metabolic capacity of this bacterium. Finally, the specific VOCs with high concentrations produced by *Bacillus safensis* were 2,3-butanedione (21.43%), 3-pentanol, 2-methyl- (36.53%), benzaldehyde (2.17%), 2,3-butanediol (4.67%), propanoic acid, 2-methyl- (3.16%), oleic acid (4.59%), and 9,12-octadecadienoic acid (Z, Z)- (3.58%).

Additionally, the GC–MS analysis showed that all five bacterial isolates contained various fatty acids along with other volatile organic compounds. It is well-known that fatty acids and their derivatives can exhibit powerful antibacterial and antifungal activities [52,53,54,55,56,57,58,59,60,61,62,63,64,65,66,67,68]. Indeed, fatty acids are known for their biodegradability, low toxicity, and resistance to extremes in pH, salinity, and temperature, which make them environmentally friendly compounds. These properties have led to their acceptance and use as food additives in various applications. Antifungal fatty acids, particularly those found in natural sources, may have a lower likelihood of inducing resistance in pathogenic fungi compared to some synthetic antifungal drugs [84]. The identification of volatile compounds, including esters, alkaloids, ethers, and phenolics, in the five different *Bacillus* species, is noteworthy and suggests a diverse array of secondary metabolites produced by these bacteria. The presence of volatile organic compounds (VOCs) as major constituents of a bacterial strain with properties against phytopathogens is significant and highlights the potential of these bacteria for various agricultural and environmental applications. The presence of common volatile compounds among the different *Bacillus* species suggests the existence of conserved metabolic pathways or biochemical processes within the genus *Bacillus.* These shared compounds may be indicative of fundamental metabolic activities that are essential for the survival and growth of these bacteria. Our findings support prior research on the chemical composition of bacterial strains using GC–MS and show that *Bacillus* spp. share similar volatile chemicals [30,85,86,87,88].

The antibacterial characteristic of certain microorganisms, including *Bacillus* strains, plays a crucial role in various therapeutic activities. The perpendicular streak method is a common laboratory technique used to assess the antibacterial properties of bacterial isolates against selected human bacterial pathogens. In the present study, this method was used to analyze the antibacterial potency of the bacterial isolates labeled BSS25, BSS21, BSS16, BSS13, and BSS12 against selected human bacterial pathogens. This effect can be explained with the help of bioactive compounds produced by the bacteria, which can have various effects on other organisms, including antimicrobials. The perpendicular streak method is recognized as a first-pass qualitative screening method for the detection of microbial activity, particularly when assessing the antibacterial properties of bacterial isolates. The research demonstrated strong antagonistic action against human pathogens such as *Enterococcus hirae* and *Staphylococcus epidermidis* by all of the bacterial isolates. It is obvious that the synergistic contribution of antibacterial potency refers to the combined effect of two or more antimicrobial agents (such as antibiotics or other antimicrobial com-pounds) that work together to produce a stronger inhibitory or bactericidal effect against bacteria than the individual agents would achieve on their own. According the GC–MS analysis, of the 69 identified compounds, many had antibacterial potency (Table 9) and their synergetic contribution explains how all bacterial strains have shown strong antagonistic action against *Enterococcus hirae* and *Staphylococcus epidermidis.* Moreover, the current research provided indicates that bacterial isolates BSS25, BSS21, and BSS16 exhibit strong antagonistic activity against *Candida albicans* while BSS25, BSS21, and BSS13 suppress the growth or activity of *Candida krusei*. Additionally, only BSS25 and BSS21 were found to exhibit strong inhibitory effects against *Klebsiella aerogenes*. This may be due to the high concentrations of acetoin in BSS25 and BSS21 and butanoic acid, 2-methyl- in BSS16 and BSS13. For sure acetoin and butanoic acid, 2-methyl- enhances the antibacterial potency of bacterial strains along with other organic volatile compounds, peptides, etc.

It is obvious that some substances, including acetic acid, phenol, benzaldehyde, and acetoin, can be present in bacterial cells as intracellular compounds rather than just volatile substances. Bacteria produce and utilize a wide range of organic compounds as part of their metabolic processes. For instance, acetic acid is a common organic acid. Bacterial cells may contain acetic acid as a metabolic intermediate or as part of their pH regulation. Some bacteria produce acetic acid as a byproduct of their fermentation processes, and it can accumulate intracellularly [50]. Another example is phenol, which is a toxic organic compound, and certain bacteria have the ability to degrade phenol as part of their metabolic activities. These bacteria can take up phenol and break it down intracellularly as a carbon source [74]. Benzaldehyde is an aromatic aldehyde that can be produced by bacteria. It can be used as an intermediate in various biosynthetic pathways, and it may accumulate intracellularly as part of these processes [51]. Acetoin is a compound produced during the fermentation of glucose by some bacteria. It can accumulate intracellularly as an intermediate metabolite and is often associated with the production of other compounds, including diacetyl. These compounds are examples of organic molecules that can be involved in the diverse metabolic activities of bacteria. The intracellular presence of such substances is essential for the growth, energy production, and synthesis of various cellular components. They can serve as intermediates in metabolic pathways or be stored as reserve materials. Additionally, the production and accumulation of these compounds may vary between bacterial species and their specific metabolic capabilities.

These are positive results, suggesting that these bacterial isolates may have potential applications in controlling or preventing infections caused by these human pathogens. Our results showed that *Bacillus thuringiensis*, *Bacillus toyonensis*, *Bacillus acidiproducens*, *Bacillus cereus*, and *Bacillus safensis* are effective at inhibiting the growth of some multidrug-resistant bacterial strains and this is similar to the findings of our previous study [30]. Previous investigations have found that the inhibitory impact of *Bacillus* species on other microorganisms, including pathogens, can be attributed to various factors, including the pH of the growing medium and the generation of volatile chemicals. *Bacillus* species are known to produce a variety of polypeptide antibiotic substances, including bacitracin, polymyxin, gramicidin S, and tyrothricin. These antibiotic substances have shown effectiveness against a wide range of bacteria, including both Gram-positive and Gram-negative bacteria [88].

Bacterial extract investigation is a versatile and interdisciplinary field with far-reaching implications for science, medicine, agriculture, and industry. It involves the discovery and characterization of compounds and biological activities that can address various challenges and opportunities in these domains. Bacterial extracts are screened to discover novel antibiotic compounds, and antimicrobial peptides (AMPs) with potential therapeutic applications, as bacteria produce a wide variety of substances. Moreover, bacterial extracts can be a source of potential drug candidates for the treatment of various diseases, including infectious diseases and cancer. According to recent studies, bacterial extracts are also used in bioassays to evaluate the biological activity of compounds, including screening for enzyme inhibitors or activators while other studies have proven the value of the use of bacterial extracts in agriculture to manage plant diseases and pests. For example, *Bacillus* species are known for their significant roles in agriculture and biotechnology, primarily due to their ability to produce various bioactive compounds that can benefit plant health and promote agricultural sustainability [89,90]. Moreover, bacterial extracts are analyzed to identify probiotic strains with potential benefits for gut health.

Molecular investigations can provide valuable insights into the taxonomy and genetic relatedness of different bacterial isolates and our results revealed the taxonomy of five different isolated species of *Bacillus*, which are *Bacillus thuringiensis* F3, *Bacillus toyonensis* FORT 102, *Bacillus acidiproducens* NiuFun, *Bacillus cereus* WAB2133, and *Bacillus safensis* AS-08. It was determined that the five most viable candidates of bacterial isolates BSS25, BSS21, BSS16, BSS13, and BSS12 belong to *Bacillus thuringiensis* F3 (99%), *Bacillus toyonensis* FORT 102 (99%), *Bacillus acidiproducens* NiuFun (99%), *Bacillus cereus* WAB2133 (99%), and *Bacillus safensis* AS-08 (99%), respectively, based on top hit sequence similarity results and phylogenetic analysis.

The identification of the five separate bacterial strains (*Bacillus thuringiensis*, *Bacillus toyonensis*, *Bacillus acidiproducens*, *Bacillus cereus*, and *Bacillus safensis*) and their antibacterial activity can significantly facilitate microbial screening and the isolation of active metabolites, especially against multidrug-resistant strains. Consequently, knowing the specific strains that exhibit antibacterial activity allows for targeted screening of these strains against multidrug-resistant bacterial strains. This targeted approach saves time and resources compared to the screening of a wide range of microorganisms. As the antibacterial activity is confirmed, the isolation and purification of bioactive metabolites from these strains can be prioritized. This is crucial for identifying the specific compounds responsible for the antibacterial effects. Moreover, the isolated bioactive compounds may have potential as antibiotic adjuvants, especially against multidrug-resistant strains. This can be a valuable contribution to the fight against antibiotic resistance. Additionally, these compounds may serve as lead compounds for drug discovery efforts, where further modifications or structural optimization can be performed to enhance their efficacy and reduce potential side effects. Overall, the identification of metabolites from bacterial strains and evaluation of their antibacterial and antibiotic activity provides a strong foundation for focused research and applications in various fields.

## 4. Materials and Methods

### 4.1. Isolation of Potential Strains of the Genus Bacillus spp.

Five *Bacillus* strains (BSS25, BSS21, BSS16, BSS13, and BSS12) were isolated from different vegetable samples such as tomato, potato, and carrot. A vegetable sample with a mass equal to 15 g was homogenized in a solvent with a volume of 100 mL of NaCl by shaking at 150 rpm for 20 min. Then, the sample was steadily diluted and incubated for 10 min at 90 °C. After the incubation the sample was cooled to room temperature. The sample with a volume of 0.1 mL was loaded onto nutrient agar/meat peptone agar (NA/MPA) plates, which serve as a fertile medium for the growth of undemanding microorganisms. The NA/MPA plates consisted of bacteriological agar (BA, 15 g/L), gelatin peptone (GP, 5 g/L), and meat extract (ME, 3 g/L). The plates were incubated at 37 °C for 48 h. The isolated pure strains were refrigerated at −20 °C in nutrient broth (NB) media supplemented with 20% (*v*/*v*) glycerin. Then, morphological identification was performed on the newly created culture. Slightly raised, flat, white, and cream-colored colonies were chosen for further study. Strain isolates were helpful in further studies, particularly in the preparation of ethyl acetate extract for subjecting GC–MS analysis.

### 4.2. Antagonistic Activity Study

On Mueller–Hinton agar (MHA) plates, a preliminary antibacterial investigation of the isolates was carried out using the perpendicular streak method against potent human pathogens. Overall, *n* = 15 bacterial pathogens were used in the antagonistic activity study: *Salmonella enterica* ATCC 35664, *Klebsiella aerogenes* ATCC 13048, *Serratia marcescens* ATCC 13880, *Klebsiella pneumoniae* ATCC 13883, *Streptococcus* group B, *Escherichia coli* ATCC 25922, *Candida krusei* ATCC 14243, *Shigella sonnei* ATCC 25931, *Streptococcus mutans* ATCC 25175, *Enterococcus hirae* ATCC 10541, *Proteus Vulgaris* ATCC 6380, *Staphylococcus epidermidis* ATCC 12228, *Pseudomonas aeruginosa* ATCC 9027, *Staphylococcus aureus* ATCC 29213, and *Candida albicans* ATCC 2091. On the basis of the perpendicular streak method, an exponential culture of the studied pathogens was streaked on the surface of an agar medium and incubated at 30 ± 4 °C for 24 h [91]. Then, an exponential culture of the test strain was inoculated perpendicularly from the edge of the cup to the stroke of the grown culture of the antagonist with a stroke by slightly touching the stroke of the antagonist strain. The plate was once more incubated in a setting that encouraged test culture growth. The samples were then processed using the first technique.

The second method, an agar well-diffusion method with a few minor modifications, was used to measure antimicrobial activity [92]. On the plate, bacterial suspensions were applied with turbidity that was calibrated to the McFarland 0.5 standard (about 108 colony forming units, or CFU per milliliter). Using the back end of a sterile 1-mL pipette tip, a 7 mm diameter well was punched aseptically onto Mueller–Hinton agar (Oxoid, Basingstoke, UK). The positive control was streptomycin (1 g/mL). Each well received 100 L of test agent in total. The diameter of the clear zone was measured during an incubation period of 16 to 24 h at 37 °C.

### 4.3. Antibiotic Susceptibility of the Bacillus Isolates

Using the disk diffusion method, the antibiotic susceptibility test of all five *Bacillus* strains (BSS25, BSS21, BSS16, BSS13, and BSS12) was carried out in accordance with the guidelines of the European Committee on Antimicrobial Susceptibility Testing (EUCAST, 2019). The choice of sterile water as the solvent was based on its ability to effectively solubilize the compounds of interest without causing any interference in the subsequent analyses. It was selected due to its compatibility with the chemical properties of the compounds. Using an aliquot of 1 mL for each strain and a concentration of 106 CFU/mL (0.5 McFarland, Hi-media, India), the *Bacillus* strains were spread-plated on Mueller–Hinton (MH) agar using sterile beads. The plates were subjected to drying for an hour. Consequently, antibiotic disks were inserted into the agar plates containing an inoculated strain. After an incubation period (24 h) at 37 °C, the widths of the inhibition zones surrounding the antibiotic disks were measured using an electronic digital vernier caliper micrometer measuring instrument (ZHHRHC LCD, Hardened, China). This helped to identify parameters such as the strain’s antibiotic susceptibility (S), intermediate resistance (I), or resistance (R) according to the CLSI guidelines (2012) [93,94]. Antibiotic disks (*n* = 18) contained a sample each of ampicillin (AMP, 10), amoxycillin (AMOX, 30), amoxycillin-clavulanic acid (AMC, 30), azithromycin (AZM, 15), bacitromycin (B, 10), carbenicillin (CAR, 100), cefepime (FEP, 30), cefepime/clavulanic acid FEC-40, cephalatin (KF, 30), cefotaxime (CTX, 30), cloxacillin (CX, 5), erythromycin (ERO, 15), gentamicin (CN, 120), polymyxin (PB, 300), penicillin G (PEN, 10), streptomycin (STR, 10), tobramycin (TOB, 10), and tetracycline (TET, 30).

### 4.4. Gas Chromatography–Mass Spectrum Analysis

Volatile substance extraction for each bacterial isolate (*Bacillus thuringiensis* F3, *Bacillus toyonensis* FORT 102, *Bacillus acidiproducens* NiuFun, *Bacillus cereus* WAB2133, and *Bacillus safensis* AS-08) was carried out separately two times from the culture broth with 25 mL ethyl acetate (Sigma-Aldrich, Hamburg, Germany) for 20 min and the two extracts were combined. After that, 1.5 mL of the extract was transferred into plastic vials with a capacity of 2 mL, which were then set on the autosampler tray for GC-MS analysis. Bacterial secondary metabolites were examined through a GC-MS analysis on a Thermo Scientific (Waltham, MA, USA) GC Focus Series DSQ. Helium was used as the carrier gas at a constant flow rate of 1 mL per minute and the injection volume of the sample was equal to 1 mL. The injector and hot oven were kept at 250 °C and 110 °C, respectively, with the temperature increasing by 10 °C per minute up to 200 °C, by 5 °C per minute up to 280 °C, and shutting down after 9 min at a temperature of 280 °C [30]. The retention durations of several chemical peaks that were eluted from the GC column were recorded. After matching the data with the mass spectra of the compounds, the database was searched for compounds with comparable molecular masses and retention times. The current investigation discovered a parallel pattern in the bioactivities of previously studied natural compounds.

### 4.5. Molecular Characterization of the Bacterial Isolates

Isolated bacterial strains were molecularly characterized using universal bacterial primers and 16S rRNA conserved gene sequences. Using the conventional PCR method, the targeted gene sequence was amplified. The final result was then processed via 1% gel electrophoresis to determine the size of the amplified fragments. The amplified materials were delivered for sequencing along with the pertinent sequencing fragments. The nucleotide sequences were phylogenetically analyzed using MEGA software (MEGA-11). The bacterial isolates were subsequently validated and categorized at the species level using GenBank NCBI’s BLAST search (National Center for Biotechnology Information). The accession numbers (MF135173, MG561363, MF446886, MH169322, and JX849661) correspond to the 16S rRNA gene sequences of the probiotic strains and were used to retrieve and reference the sequences in the GenBank database. (www.ncbi.nlm.nih.gov/projects/genome/clone/, accessed on 9 July 2023).

### 4.6. Statistical Analysis

The XLSAT software, version 2016.02.27444, was used to perform the one-factor analysis of variance at the significance level (α = 0.05). The Newman–Keuls test was used to rank the means when there were substantial differences between the parameters under study.

## 5. Conclusions

In the current study, vegetable bacterial isolates obtained from five different species of *Bacillus* demonstrated the ability to inhibit the growth of multidrug-resistant bacterial strains. As a result of a screening process, five potent isolates named BSS25, BSS21, BSS16, BSS13, and BSS12 were identified. GC–MS was used to identify and quantify the chemical compounds present in the bacterial species, despite the fact that they are all members of the same *Bacillus* subspecies. The observation was that volatile organic compounds (VOCs) differ among members of the same *Bacillus* subspecies despite their taxonomic similarity, highlighting the chemical diversity that can exist within closely related bacterial strains. This study confirmed a variety of volatile inhibitory substances, including esters, phenolics, and ethers, which are believed to play a role in antimicrobial activity. The volatile compounds differ in chemical composition among the tested samples suggesting that these variations may have an impact on antimicrobial activity and antibiotic potency. Strains of the Bacillus subtilis group are known to have the capability to produce a wide range of secondary metabolites that contribute to their antimicrobial characteristics and this was also confirmed by our findings. In addition to volatile organic compounds, strains within the *Bacillus* subtilis group are known to produce a variety of other bioactive compounds, including bacteriocins, polyketides, peptides, and more. Hence, it can be concluded that the discovered organic volatile substances, i.e., acetoin and butanoic acid, 2-methyl- enhance the antimicrobial properties of *Bacillus* spp. together with the above substances. The remarkable metabolic capacity and adaptive biochemistry of *Bacillus* species, i.e., *Bacillus thuringiensis* (BSS25), *Bacillus toyonensis* (BSS21), *Bacillus acidiproducens* (BSS16), *Bacillus cereus* (BSS13), and *Bacillus safensis* (BSS12) make these strains valuable for various commercial and biotechnological applications, as they have the potential to generate a wide range of bioactive chemical substances. Bacterial extracts, which contain bioactive chemicals produced by these *Bacillus* strains, have the potential to be used as antimicrobial agents. The anticipation of conducting a thorough investigation, similar to the one described, holds great promise for uncovering new microbiological possibilities and discovering previously unknown substances or metabolites with strong antibacterial potential. Such research endeavors are essential for addressing the burden and danger posed by bacterial strains that have developed resistance to multiple drugs.

## Figures and Tables

**Table 1:** Colony morphology and microscopic presentation of isolated bacterial species.

Bacterial Species	Media	Colony Color and Texture	Microscopic Presentation
*Bacillus thuringiensis* F3 (BSS25)	*Bacillus* Medium.	White, irregular, flat.	Gram-strain-positive, spore-forming, rod.
*Bacillus toyonensis* FORT 102 (BSS21)	*Bacillus* Medium.	White, irregular, flat.	Gram-strain-positive, spore-forming, rod.
*Bacillus acidiproducens* NiuFun (BSS16)	*Bacillus* Medium.	White, irregular, flat.	Gram-strain-positive, spore-forming, rod.
*Bacillus cereus* WAB2133 (BSS13)	*Bacillus* Medium.	White, irregular, flat.	Gram-strain-positive, spore-forming, rod.
*Bacillus safensis* AS-08 (BSS12)	*Bacillus* Medium.	White, irregular, flat.	Gram-strain-positive, spore-forming, rod.

**Table 2:** Antibacterial activity of the bacterial culture extracts against pathogenic strains.

Species of Microorganism	BSS25	BSS21	BSS16	BSS13	BSS12	Control (Streptomycin)
*Staphylococcus aureus* ATCC 29213	35 ± 1.27	9 ± 1.53 *	23 ± 0.50 *	20 ± 2.50 *	20 ± 1.54	24 ± 0.33 ***
*Staphylococcus epidermidis* ATCC 12228	37 ± 1.47	36 ± 1.27	38 ± 1.27	37 ± 1.07	35 ± 1.47	22 ± 0.33 ***
*Streptococcus group B*	18 ± 1.56 *	19 ± 1.31 *	19 ± 1.23 *	18 ± 1.56 *	17 ± 1.39 *	17 ± 0.33 ***
*Streptococcus mutans* ATCC 25175	20 ± 1.47 *	19 ± 1.27 *	23 ± 1.33	20 ± 1.33	20 ± 1.37 *	19 ± 0.33 ***
*Candida albicans* ATCC 2091	36 ± 1.,43	38 ± 1.21	38 ± 1.27	35 ± 1.26	34 ± 1.22	31 ± 0.33 ***
*Candida krusei* ATCC 14243	37 ± 1.41	36 ± 1.28	8 ± 1.38 *	36 ± 1027	13 ± 1.27 *	30 ± 0.33 ***
*Pseudomonas aeruginosa* ATCC 9027	18 ± 0.53 *	17 ± 1.27 *	17 ± 0.33 *	17 ± 1.10 *	16 ± 1.33 *	15 ± 0.33 ***
*Shigella sonnei* ATCC 25931	20 ± 1.27 *	33 ± 1.37 *	21 ± 1.57 *	21 ± 1.37 *	21 ± 1.06 *	19 ± 0.33 ***
*Klebsiella pneumonia* ATCC 13883	9 ± 1.53 *	9 ± 1.27 *	8 ± 1.27 *	8 ± 1.37 *	8 ± 1.37 *	12 ± 0.33 ***
*Salmonella enterica* ATCC 35664	17 ± 1.36 *	15 ± 1.25 *	18 ± 1.27 *	15 ± 1.27 *	17 ± 1.27 *	19 ± 0.33 ***
*Klebsiella aerogenes* ATCC 13048	37 ± 1.27	36 ± 1.37	32 ± 1.33 *	35 ± 0.63 *	33 ± 1.33 *	23 ± 0.33 ***
*Enterococcus hirae* ATCC 10541	37 ± 1.25	38 ± 1.27	38 ± 1.27	39 ± 1.27	38 ± 1.27	22 ± 0.33 ***
*Escherichia coli* ATCC 25922	17 ± 1.37 *	16 ± 1.07 *	19 ± 1.44 *	18 ± 1.33 *	20 ± 1.08 *	16 ± 0.33 ***
*Serratia marcescens* ATCC 13880	27 ± 0.56 *	29 ± 1.36 *	25 ± 1.32 *	28 ± 0.53 *	27 ± 1.53 *	22 ± 0.33 ***
*Proteus vulgaris* ATCC 6380	20 ± 0.31 *	19 ± 1.31 *	21 ± 1.33 *	20 ± 1.23 *	21 ± 0.33 *	22 ± 0.33 ***

Data are represented as means ± SE (*n* = 3). Values with the same superscript symbols are not statistically different. Significance level * < ***.

**Table 3:** Antibiotic resistance profile of the *Bacillus* strains.

Antibiotic (AB, Charge in μg) Used	*Bacillus* Strains									
	BSS25		BSS21		BSS16		BSS13		BSS12	
	Diameter (mm)	S/R	Diameter (mm)	S/R	Diameter (mm)	S/R	Diameter (mm)	S/R	Diameter (mm)	S/R
**Penicillins:**										
Penicillin G (PEN, 10)	37± 0.19 ^a^	**S**	20 ± 0.35 ^ab^	**S**	34 ± 0.48 ^abc^	**S**	22 ± 1.43 ^a^	**S**	22 ± 1.43 ^a^	**S**
Ampicillin (AMP, 10)	30 ± 0.21 ^ab^	**S**	20 ± 0.35 ^ab^	**S**	36 ± 0.36 ^ab^	**S**	32 ± 0.98 ^ab^	**S**	32 ± 0.98 ^ab^	**S**
Amoxycillin (AMOX, 30)	40 ± 0.21 ^a^	**S**	20 ± 0.35 ^ab^	**S**	38 ± 0.41 ^abc^	**S**	35 ± 1.81 ^abc^	**S**	35 ± 1.81 ^abc^	**S**
Amoxycillin-clavulanic acid (AMC, 30)	35 ± 0.98 ^c^	**S**	20 ± 0.35 ^abc^	**S**	36 ± 0.98 ^abc^	**S**	30 ± 1.45 ^ab^	**S**	30 ± 1.45 ^b^	**S**
Carbenicillin (CAR, 100)	35 ± 0.32 ^abs^	**S**	20 ± 0.35 ^ab^	**S**	34 ± 0.56 ^abc^	**S**	35 ± 1.43 ^abc^	**S**	35 ± 1.43 ^abc^	**S**
Cloxacillin (CX, 5)	14 ± 0.23 ^a^	**R**	14 ± 0.23 ^a^	**R**	14 ± 0.23 ^ab^	**R**	14 ± 0.23 ^abc^	**R**	14 ± 0.23 ^a^	**R**
**Macrolides:**										
Erythromycin (ERO, 15)	30 ± 0.21 ^a^	**S**	20 ± 0.11 ^abc^	**S**	40 ± 0.39 ^ab^	**S**	40 ± 0.28 ^a^	**S**	30 ± 0.21 ^a^	**S**
Azithromycin (AZM, 15)	30 ± 0.22 ^a^	**S**	20 ± 0.35 ^a^	**S**	35 ± 0.59 ^ab^	**S**	37 ± 1.52 ^a^	**S**	30 ± 0.22 ^a^	**S**
**Cephalosporins:**										
Cefepime (FEP, 30)	30 ± 0.35 ^bcd^	**S**	30 ± 0.35 ^abc^	**S**	30 ± 0.36 ^ab^	**S**	20 ± 1.43 ^ab^	**S**	20 ± 1.43 ^ab^	**S**
Cefepime/clavulanic acid FEC-40	30 ± 0.35 ^a^	**S**	30 ± 0.35 ^ab^	**S**	33 ± 0.28 ^a^	**S**	26 ± 0.23 ^a^	**S**	26 ± 0.23 ^a^	**S**
Cephalatin (KF, 30)	34 ± 0.21 ^ab^	**S**	30 ± 0.35 ^a^	**S**	30 ± 0.54 ^a^	**S**	25 ± 0.98 ^ab^	**S**	25 ± 0.98 ^abs^	**S**
Cefotaxime (CTX, 30)	27 ± 0.35 ^a^	**S**	28 ± 0.11 ^a^	**S**	25 ± 0.28 ^a^	**S**	35 ± 1.29 ^ab^	**S**	35 ± 1.29 ^a^	**S**
**Aminoglycosides:**										
Gentamicin (CN, 120)	39 ± 0.37 ^ab^	**S**	38 ± 0.43 ^ab^	**S**	40 ± 0.12 ^ab^	**S**	41 ± 0.23 ^ab^	**S**	39 ± 0.31 ^ab^	**S**
Streptomycin (STR, 10)	23 ± 0.36 ^ab^	**S**	25 ± 0.31 ^ab^	**S**	28 ± 1.41 ^ab^	**S**	23 ± 1.41 ^ab^	**S**	25 ± 0.31 ^ab^	**S**
Tobramycin (TOB, 10)	32 ± 0.32 ^a^	**S**	25 ± 0.31 ^a^	**S**	34 ± 1.18 ^a^	**S**	35 ± 0.98 ^ab^	**S**	35 ± 1.29 ^a^	**S**
**Tetracyclines:**										
Tetracycline (TET, 30)	30 ± 0.52 ^a^	**S**	26 ± 0.15 ^a^	**S**	36 ± 1.43 ^a^	**S**	30 ± 0.23 ^a^	**S**	30 ± 0.52 ^ab^	**S**
**Polypeptides:**										
Polymyxin (PB, 300)	7 ± 0.28 ^b^	**R**	0 ± 0.00 ^b^	**R**	8 ± 1.49 ^b^	**R**	10± 0.23 ^b^	**R**	0 ± 0.00 ^b^	**R**
Bacitromycin (B, 10)	0 ± 0.00 ^b^	**R**	0 ± 0.00 ^b^	**R**	0 ± 0.00 ^b^	**R**	0 ± 0.00 ^b^	**R**	0 ± 0.00 ^b^	**R**

The Newman–Keuls test was used to compare means in a data set, and the results suggest that there are statistically significant differences among some of the groups. Here, “±” is a standard error; legend: D and S/R are dimension and sensible/resistant, respectively. Different letters of “a”, “b”, “c”, “d” and “s” indicate statistical differences at *p* < 0.05.

**Table 4:** The main constituents of bacterial extract BSS25 were identified through GC–MS analysis.

*Bacillus thuringiensis* (BSS25)							
No.	Name	Molecular Formula	Molecular Mass, g/mol	Retention Time (min)	PubChem Compound CID	Similarities	Area, %
1	Acetone	C_3_H_6_O	58.08	1.642	180	87	0.17
2	2,3-Butanedione	C_4_H_6_O	86.09	2.64	650	93	15.85
3	Hexanal	C_6_H_12_O	100.16	3.617	6184	65	0.34
4	Acetoin	C_4_H_8_O_2_	88.11l	6.239	179	89	44.06
5	3-Pentanol, 2-methyl-	C_6_H_14_O	102.17	7.012	11,264	80	1.37
6	Oxirane, (methoxymethyl)-	C_4_H_8_O_2_	88.11	7.219	13,589	79	1.36
7	Acetic acid	C_2_H_4_O_2_	60.05	8.368	176	86	0.89
8	Decanal	C_10_H_20_O	156.26	8.504	8175	73	0.39
9	1-Hexanol, 2-ethyl-	C_8_H_18_O	130.229	8.896	7720	87	0.24
10	Benzaldehyde	C_7_H_6_O	106.12	9.411	240	94	2.22
11	(R,R)-2,3-Butanediol	C_4_H_10_O	90.12	9.491	439,888	82	4.08
12	1,6-Octadien-3-ol, 3,7-dimethyl-	C_10_H_18_O	154.25	9.64	6549	85	0.78
13	1-Hepten-4-ol	C_7_H_14_O	114.19	9.847	19,040	70	3.42
14	1-Nonanol	C_9_H_20_O	144.25	10.47	8914	78	0.73
15	(S)-(+)-6-Methyl-1-octanol	C_9_H_20_O	144.25	10.639	13,548,104	85	1.25
16	Butanoic acid, 2-methyl-	C_5_H_10_O_2_	102.13	11.091	8314	81	2.24
17	Oxime-, methoxy-phenyl	C_8_H_9_NO_2_	151.16	12.063	9,602,988	73	1.136
18	1-Decanol	C_10_H_22_O	158.28	12.21	8174	60	0.52
19	Hexanoic acid	C_6_H_12_O_2_	116.16	13.085	8892	91	1.86
20	5,9-Undecadien-2-one, 6,10-dimethyl-, (E)-	C_13_H_22_O	194.31	13.329	1,549,778	60	0.66
21	Propanoic acid, 2-methyl-, 3-hydroxy-2,4,4-trimethylpentyl ester	C_12_H_24_O	216.32	13.462	551,387	65	0.99
22	2,2,4-Trimethyl-1,3-pentanediol diisobutyrate	C_16_H_30_O_4_	286.41	13.63	23,284	81	0.91
23	(R)-(−)-4-Methylhexanoic acid	C_7_H_14_O_2_	130.18	13.965	12,600,623	70	0.62
24	Hexanoic acid, 2-ethyl-	C_8_H_16_O_2_	144.21	14.226	8697	89	2.90
25	Cetene	C_16_H_32_	224.42	14.477	12,395	81	0.75
26	Phenol	C_6_H_6_O	94.11	14.825	996	89	0.82
27	Neodecanoic acid	C_10_H_20_O_2_	172.26	15.305	62,838	61	0.43
28	Octanoic acid	C_8_H_16_O_2_	144.21	15.386	379	91	2.47
29	1,2-Benzenedicarboxylic acid, bis(2-methylpropyl) ester	C_16_H_22_O_4_	278.34	16.151	6782	77	0.44
30	Nonanoic acid	C_9_H_18_O_2_	158.24	16.478	8158	90	2.80
31	Benzoic acid, 2-ethylhexyl ester	C_15_H_22_O_2_	234.33	17.083	94,310	61	0.17
32	Hexadecanoic acid, methyl ester	C_17_H_34_O_2_	270.5	17.161	8181	91	1.22
33	2-Octyl benzoate	C_15_H_22_O_2_	234.33	17.531	243,800	69	0.90

**Table 5:** The main constituents of bacterial extract BSS21 were identified through GC–MS analysis.

*Bacillus toyonensis* (BSS21)							
No.	Name	Molecular Formula	Molecular Mass, g/mol	Retention Time (min)	PubChem Compound CID	Similarities	Area, %
1	Acetone	C_3_H_6_O	58.08	1.661	180	79	0.55
2	2,3-Butanedione	C_4_H_6_O_2_	86.09	2.652	650	92	16.97
3	2,3-Pentanedione	C_5_H_8_O_2_	100.12	3.404	11,747	68	2.035
4	Acetoin	C_4_H_8_O_2_	88.11	6.246	179	89	38.25
5	3-Pentanol, 2-methyl-	C_6_H_14_O	102.17	7.008	11,264	81	2.92
6	Oxirane, (methoxymethyl)-	C_4_H_8_O_2_	88.11	7.216	13,589	80	2.41
7	Nonanal	C_9_H_18_O	142.24	7.73	31,289	85	0.75
8	Acetic acid	C_2_H_4_O_2_	60.05	8.343	176	97	2.46
9	1-Hexanol, 2-ethyl-	C_8_H_18_O	130.229	8.889	7720	88	0.15
10	E-3-Pentadecen-2-ol	C_15_H_30_O	226.4	9.048	5,363,322	65	0.14
11	(R,R)-2,3-Butanediol	C_4_H_10_O_2_	90.12	9.486	439,888	88	7.80
12	Formic acid, octyl ester	C_9_H_18_O_2_	158.24	9.751	8176	69	0.37
13	Propanoic acid, 2-methyl-	C_4_H_8_O_2_	88.11	9.832	6590	76	1.40
14	2-Octanol	C_8_H_18_O	130.229	9.928	20,083	74	0.26
15	(S)-(+)-6-Methyl-1-octanol	C_9_H_20_O	144.25	10.631	13,548,104	82	0.28
16	1-Nonanol	C_9_H_20_O	144.25	11.003	8914	78	0.13
17	Butanoic acid, 2-methyl-	C_5_H_10_O_2_	102.13	11.073	8314	87	3.57
18	Dodecanal	C_12_H_24_O	184.32	11.714	8194	92	0.68
19	Oxime-, methoxy-phenyl-_	C_8_H_9_NO_2_	151.16	12.052	9,602,988	73	0.35
20	2,4-Decadienal, (E, E)-	C_10_H_16_O	152.23	12.851	5,283,349	77	0.28
21	Pentanoic acid	C_5_H_10_O_2_	102.13	13.071	7991	79	0.23
22	3-Buten-2-one, 4-(1-cyclopenten-1-yl)-, (E)-	C_9_H_12_O	136.19	13.461	5,370,075	76	0.40
23	Hexanoic acid, 2-ethyl-	C_8_H_16_O_2_	144.21	14.199	8697	80	0.31
24	1-Dodecanol	C_12_H_26_O	186.33	14.446	8193	81	0.15
25	Phenol	C_6_H_6_O	94.11	14.774	996	90	0.19
26	Octanoic acid	C_8_H_16_O_2_	144.21	15.314	379	79	0.40
27	Nonanoic acid	C_9_H_18_O_2_	158.24	16.359	8158	88	0.73
28	Hexadecanoic acid, methyl ester	C_17_H_34_O_2_	270.5	17.01	8181	88	0.32
29	1,4-Benzenediol, 2,6-bis(1,1-dimethylethyl)-	C_14_H_22_O_2_	222.32	17.298	75,550	63	0.18
30	Decanoic acid	C_10_H_20_O_2_	172.26	17.356	2969	64	0.31
31	Benzoic acid, heptyl ester	C_14_H_20_O_2_	220.31	18.369	81,591	73	0.16
32	Benzoic acid	C_7_H_6_O_2_	122.12	18.739	243	85	0.22
33	1,2-Benzenedicarboxylic acid, bis(2-methylpropyl) ester	C_16_H_22_O_4_	278.34	19.85	6782	81	0.33
34	Dibutyl phthalate	C_16_H_22_O_4_	278.34	21.099	3026	67	0.32
35	Hexadecanoic acid	C_16_H_32_O_2_	256.42	22.611	985	76	4.00
36	Oleic Acid	C_18_H_34_O_2_	282.5	24.54	445,639	86	5.85
37	9,12-Octadecadienoic acid (Z, Z)-	C_18_H_32_O_2_	280.4	25.063	5,280,450	87	3.80

**Table 6:** The main constituents of bacterial extract BSS16 were identified through GC–MS analysis.

*Bacillus acidiproducens* (BSS16)							
No.	Name	Molecular Formula	Molecular Mass, g/mol	Retention Time (min)	PubChem Compound CID	Similarities	Area, %
1	Acetone	C_3_H_6_O	58.08	1.67	180	93	0.75
2	Acetoin	C_4_H_8_O_2_	88.11	6.49	179	89	8.44
3	Acetic acid	C_2_H_4_O_2_	60.05	8.363	176	95	2.40
4	Benzaldehyde	C_7_H_6_O	106.12	9.411	240	94	4.75
5	3(2H)-Thiophenone, dihydro-2-methyl-	C_5_H_8_OS	116.18	9.463	61,664	84	6.07
6	Propanoic acid, 2-methyl-	C_4_H_8_O_2_	88.11	9.839	6590	92	13.97
7	Butanoic acid	C_4_H_8_O_2_	88.11	10.581	264	83	0.61
8	Butanoic acid, 2-methyl-	C_5_H_10_O_2_	102.13	11.084	8314	83	29.39
9	Oxime-, methoxy-phenyl-_	C_8_H_9_NO_2_	151.16	12.063	9,602,988	76	1.14
10	Phenol	C_6_H_6_O	94.11	14.788	996	90	0.32
11	Nonanoic acid	C_9_H_18_O_2_	158.24	16.372	8158	85	0.54
12	Hexadecanoic acid, methyl ester	C_17_H_34_O_2_	270.5	17.016	8181	91	0.72
13	2-Octyl benzoate	C_15_H_22_O_2_	234.33	17.363	243,800	68	0.68
14	Benzoic acid 2-methylpentyl ester	C_13_H_18_O_2_	206.28	17.813	570,433	66	0.53
15	Benzoic acid, heptyl ester	C_14_H_20_O_2_	220.31	18.084	81,591	80	0.50
16	Benzoic acid, tridecyl ester	C_20_H_32_O_2_	304.5	18.375	9,814,973	75	0.56
17	Benzoic acid	C_7_H_6_O_2_	122.12	18.752	243	79	0.65
18	1,2-Benzenedicarboxylic acid, bis(2-methylpropyl) ester	C_16_H_22_O_4_	278.34	19.859	6782	91	0.94
19	Pentadecanoic acid	C_15_H_30_O_2_	242.4	21.427	13,849	63	0.41
20	Hexadecanoic acid	C_16_H_32_O_2_	256.42	22.604	985	88	3.60
21	Octadecanoic acid	C_18_H_36_O_2_	284.5	24.256	5281	72	1.64
22	Oleic acid	C_18_H_34_O_2_	282.5	24.542	445,639	90	9.68
23	9,12-Octadecadienoic acid (Z, Z)-	C_18_H_32_O_2_	280.4	25.067	5,280,450	91	11.10

**Table 7:** The main constituents of bacterial extract BSS13 were identified through GC–MS analysis.

*Bacillus cereus* (BSS13)							
No.	Name	Molecular Formula	Molecular Mass, g/mol	Retention Time (min)	PubChem Compound CID	Similarities	Area, %
1	Acetone	C_3_H_6_O	58.08	1.644	180	94	3.66
2	Acetoin	C_4_H_8_O_2_	88.11	6.49	179	89	0.74
3	Acetic acid	C_2_H_4_O_2_	60.05	8.335	176	97	6.31
4	Decanal	C_10_H_20_O	156.26	9.114	8175	70	0.63
5	Benzaldehyde	C_7_H_6_O	106.12	9.402	240	95	6.24
6	Propanoic acid, 2-methyl-	C_4_H_8_O_2_	88.11	9.828	6590	92	11.51
7	Butanoic acid	C_4_H_8_O_2_	88.11	10.569	264	86	0.91
8	Butanoic acid, 2-methyl-	C_5_H_10_O_2_	102.13	11.081	8314	83	31.69
9	Oxime-, methoxy-phenyl-_	C_8_H_9_NO_2_	151.16	12.054	9,602,988	67	1.28
10	Tiglic acid	C_5_H_8_O_2_	100.12	13.078	125,468	81	1.68
11	(R)-(−)-4-Methylhexanoic acid	C_7_H_14_O_2_	130.18	13.945	12,600,623	84	0.53
12	Hexanoic acid, 2-ethyl-	C_8_H_16_O_2_	144.21	14.197	8697	88	1.03
13	Phenol	C_6_H_6_O	94.11	14.777	996	87	0.44
14	Octanoic acid	C_8_H_16_O_2_	144.21	15.317	379	90	1.11
15	Nonanoic acid	C_9_H_18_O_2_	158.24	16.362	8158	90	2.40
16	Hexadecanoic acid, methyl ester	C_17_H_34_O_2_	270.5	17.016	8181	80	1.09
17	Decanoic acid	C_10_H_20_O_2_	172.26	17.359	2969	66	0.94
18	Benzoic acid 2-methylpentyl ester	C_13_H_18_O_2_	206.28	17.809	570,433	64	0.55
19	Benzoic acid, heptyl ester	C_14_H_20_O_2_	220.31	18.079	81,591	78	0.42
20	Benzoic acid	C_7_H_6_O_2_	122.12	18.742	243	87	0.57
21	1,2-Benzenedicarboxylic acid, bis(2-methylpropyl) ester	C_16_H_22_O_4_	278.34	19.852	6782	93	1.11
22	Dibutyl phthalate	C_16_H_22_O_4_	278.34	21.116	3026	69	0.94
23	Hexadecanoic acid	C_16_H_32_O_2_	256.42	22.596	985	84	4.45
24	Octadecanoic acid, 2-hydroxy-1,3-propanediyl ester	C_39_H_76_O_5_	625	24.244	101,269	67	3.79
25	9-Octadecenoic acid, (E)-	C_18_H_34_O_2_	282.5	24.53	637,517	86	9.95
26	9,12-Octadecadienoic acid (Z, Z)-	C_18_H_32_O_2_	280.4	25.052	5,280,450	83	5.87

**Table 8:** The main constituents of bacterial extract BSS12 were identified through GC–MS analysis.

*Bacillus safensis* (BSS12)							
No.	Name	Molecular Formula	Molecular Mass, g/mol	Retention Time (min)	PubChem Compound CID	Similarities	Area, %
1	(2-Aziridinylethyl)amine	C_4_H_10_N_2_	86.14	1.162	97697	96	0.39
2	1-Propen-2-ol, acetate	C_5_H_8_O_2_	100.12	1.667	7916	65	0.78
3	2,3-Butanedione	C_4_H_6_O_2_	86.09	2.647	650	93	21.43
4	3-Penten-1-ol	C_5_H_10_O	86.13	3.411	510,370	69	2.64
5	Acetoin	C_4_H_8_O_2_	88.11	5.703	179	89	0.26
6	3-Pentanol, 2-methyl-	C_6_H_14_O	102.17	6.259	11,264	72	36.53
7	2-Nonen-1-ol	C_9_H_18_O	142.24	7.011	61,896	82	1.23
8	2-Hydroxy-3-pentanone	C_5_H_10_O_2_	102.13	7.109	521,790	73	0.42
9	Ethane-1,1-diol dibutanoate	C_10_H_18_O_4_	202.25	7.215	551,339	83	1.18
10	Acetic acid	C_2_H_4_O_2_	60.05	8.354	176	90	0.78
11	1-Hexanol, 2-ethyl-	C_8_H_18_O	130.229	8.888	7720	93	0.44
12	Benzaldehyde	C_7_H_6_O	106.12	9.397	240	96	2.17
13	2,3-Butanediol	C_4_H_10_O_2_	90.12	9.484	262	89	4.67
14	1,6-Octadien-3-ol, 3,7-dimethyl-	C_10_H_18_O	154.25	9.632	6549	87	0.78
15	Propanoic acid, 2-methyl-	C_4_H_8_O_2_	88.11	9.832	6590	67	3.16
16	(R,R)-2,3-Butanediol	C_4_H_10_O_2_	90.12	9.925	225,936	74	0.53
17	1-Nonanol	C_9_H_20_O	144.25	10.366	8914	82	0.42
18	(S)-(+)-6-Methyl-1-octanol	C_9_H_20_O	144.25	10.635	13,548,104	89	0.98
19	Butanoic acid, 2-methyl-	C_5_H_10_O_2_	102.13	11.082	8314	82	1.89
20	Oxime-, methoxy-phenyl-_	C_8_H_9_NO_2_	151.16	12.053	9,602,988	67	0.52
21	2,4-Decadienal	C_10_H_16_O	152.23	12.853	5,283,349	79	0.32
22	2,2,4-Trimethyl-1,3-pentanediol diisobutyrate	C_16_H_30_O_4_	286.41	13.131	23,284	82	0.17
23	(R)-(−)-4-Methylhexanoic acid	C_7_H_14_O_2_	130.18	13.315	12,600,623	62	0.13
24	Phenol	C_6_H_6_O	94.11	13.711	996	75	0.20
25	Octanoic acid	C_8_H_16_O_2_	144.21	14.194	379	76	0.18
26	Nonanoic acid	C_9_H_18_O_2_	158.24	14.587	8158	76	0.14
27	Hexadecanoic acid, methyl ester	C_17_H_34_O_2_	270.5	14.766	8181	96	2.89
28	2-Octyl benzoate	C_15_H_22_O_2_	234.33	15.31	243,800	70	0.17
29	Benzoic acid, heptyl ester	C_14_H_20_O_2_	220.31	15.742	81,591	75	0.10
30	Benzoic acid, undecyl ester	C_18_H_28_O_2_	276.4	16.355	229,159	88	0.40
31	Benzoic acid	C_7_H_6_O_2_	122.12	17.008	243	89	0.28
32	1,2-Benzenedicarboxylic acid, bis(2-methylpropyl) ester	C_16_H_22_O_4_	278.34	18.074	6782	69	0.09
33	Oleic Acid	C_18_H_34_O_2_	282.5	18.364	445,639	76	0.14
34	Dibutyl phthalate	C_16_H_22_O_4_	278.34	19.845	3026	89	0.32
35	Hexadecanoic acid	C_16_H_32_O_2_	256.42	22.582	985	82	2.38
36	Octadecanoic acid	C_18_H_36_O_2_	284.5	24.235	5281	69	2.67
37	Oleic Acid	C_18_H_34_O_2_	282.5	24.517	445,639	87	4.58
38	9,12-Octadecadienoic acid (Z, Z)-	C_18_H_32_O_2_	280.4	25.039	5,280,450	83	3.57

**Table 9:** The specific compounds found in high concentrations in the bacterial extracts.

	*Bacillus* spp.				
	* Bacillus thuringiensis *	* Bacillus toyonensis *	* Bacillus acidiproducens *	* Bacillus cereus *	* Bacillus safensis *
The specific compounds found in high concentrations in bacterial extracts	acetoin (44.06%); 2,3-butanedione (15.85%); (R,R)- 2,3-butanediol(4.08%); 1-Hepten-4-ol (3.42%); hexanoic acid, 2-ethyl- (2.90%); nonanoic acid (2.80%) octanoic acid (2.47%); butanoic acid, 2-methyl- (2.24%); benzaldehyde (2.22%)	acetoin (38.25%); 2,3-butanedione (16.97%); (R,R)- 2,3-butanediol (7.80%); oleic acid (5.85%); hexadecanoic acid (4.00%); 9,12-octadecadienoic acid (Z, Z)- (3.80%); butanoic acid, 2-methyl- (3.57%); 3-pentanol, 2-methyl- (2.92%); acetic acid (2.46%); oxirane, (methoxymethyl)- (2.41%); 2,3-pentanedione (2.40%)	butanoic acid, 2-methyl- (29.39%); propanoic acid, 2-methyl- (13.97%); 9,12-octadecadienoic acid (Z, Z)- (11.10%); oleic acid (9.68%); acetoin (8.44%); 3(2H)-Thiophenone, dihydro-2-methyl- (6.07%); benzaldehyde (4.75%); hexadecanoic acid (3.60%)	butanoic acid, 2-methyl- (31.69%); propanoic acid, 2-methyl- (11.51%); 9-octadecenoic acid, (E)- (9.95%); acetic acid (6.31%); benzaldehyde (6.21%); 9,12-octadecadienoic acid (Z, Z)- (5.87%); hexadecanoic acid (4.45%); octadecanoic acid, 2-hydroxy-1,3-propanediyl ester (3.79%); acetone (3.66%); nonanoic acid (2.40%); acetic acid (2.40%)	3-pentanol, 2-methyl- (36.53%); 2,3-butanedione (21.43%); 2,3-butanediol (4.67%); oleic acid (4.58%); 9,12-octadecadienoic acid (Z, Z)- (3.57%); propanoic acid, 2-methyl- (3.16%); hexadecanoic acid, methyl ester (2.89%); 3-penten-1-ol (2.64%); octadecanoic acid (2.67%); hexadecanoic acid (2.38%); benzaldehyde (2.17%)

**Table 10:** List of various classes of compounds identified from five *Bacillus* spp. and their pharmacological activities.

No.	Name	Chemical Classes	Known Pharmacological Activities
1	3-Pentanol, 2-methyl-	Alcohols	–
2	(R,R)-2,3-Butanediol	Alcohols	–
3	1,6-Octadien-3-ol, 3,7-dimethyl-	monoterpene alcohols	Anti-inflammatory, anticancer, anti-hyperlipidemic, antimicrobial, antinociceptive, analgesic, anxiolytic, anti-depressive and neuroprotective [40]
4	1-Hepten-4-ol	Alcohols	–
5	1-Nonanol	Alcohols	Antifungal [41] and antibacterial [42]
6	(S)-(+)-6-Methyl-1-octanol	Alcohols	–
7	1-Decanol	Alcohols	Antibacterial [42], antioxidant and neuroprotective [43]
8	E-3-Pentadecen-2-ol	Alcohols	–
9	2-Octanol	Alcohols	–
10	3-Penten-1-ol	Alcohols	–
11	2-Nonen-1-ol	Alcohols	–
12	2,3-Butanediol	Alcohols	CNS depressant [44], antimicrobial and antagonistic [45]
13	1-Hexanol, 2-ethyl-	Alcohols	–
14	1-Dodecanol	Alcohols	Antibacterial [42]
15	Hexanal	aldehydes	Antimicrobial [46]
16	Nonanal	aldehydes	Anti-fungal [47]
17	Decanal	aldehydes	Anti-fungal [48]
18	Dodecanal	aldehydes	–
19	2,4-Decadienal, (E,E)-	aldehydes	Flavoring agent, fragrance agent, toxic [49]
20	Acetic acid	aromatic aldehydes	Antibacterial and antifungal, anticancer [50]
21	Benzaldehyde	carboxylic acids (simple acids)	Denaturant and a flavoring agent [51]
22	Butanoic acid, 2-methyl-	carboxylic acids (simple acids)	Laxative [52]
23	Hexanoic acid	carboxylic acids (fatty acids)	–
24	(R)-(−)-4-Methylhexanoic acid	carboxylic acids (fatty acids)	–
25	Hexanoic acid, 2-ethyl-	carboxylic acids (fatty acids)	–
26	Neodecanoic acid	carboxylic acids (fatty acids)	–
27	Octanoic acid	carboxylic acids (fatty acids)	Anticancer [53], antibacterial [54], antimicrobial [55]
28	Nonanoic acid	carboxylic acids (fatty acids)	Skin-conditioning agent [56], anti-fungal [57]
29	Propanoic acid, 2-methyl-	carboxylic acids (fatty acids)	–
30	Butanoic acid	carboxylic acids (fatty acids)	The main energetic substrate of the colonocyte [58]
31	Pentadecanoic acid	carboxylic acids (fatty acids)	A JAK2/STAT3 signaling inhibitor in breast cancer cells [59], anti-biofilm agent [60]
32	Oleic Acid	carboxylic acids (fatty acids)	Anticancer, anti-inflammatory, wound healing [61]
33	Hexadecanoic acid	carboxylic acids (fatty acids)	Anti-inflammatory [62], antibacterial [63],
34	Octadecanoic acid	carboxylic acids (fatty acids)	Anticancer [64]
35	9,12-Octadecadienoic acid (Z, Z)-	carboxylic acids (fatty acids)	Used for the treatment or prevention of cardiac arrhythmias [65]
36	Tiglic acid	carboxylic acids (fatty acids)	–
37	Decanoic acid	carboxylic acids (fatty acids)	Enhances antibacterial effect [66], anti-inflammatory [67]
38	9-Octadecenoic acid, (E)-	carboxylic acids (fatty acids)	–
39	Pentanoic acid	carboxylic acids (fatty acids)	Neuroprotective agent and suppresses oxidative stress [68]
40	Acetone	ketones	Antibacterial [69]
41	2,3-Butanedione	ketones	–
42	Acetoin	ketones	CNS depressant [44]
43	5,9-Undecadien-2-one, 6,10-dimethyl-, (E)-	ketones (sesquiterpenoid)	–
44	2,3-Pentanedione	ketones	–
45	3-Buten-2-one, 4-(1-cyclopenten-1-yl)-, (E)-	ketones (cyclic)	–
46	2-Hydroxy-3-pentanone	ketones (acyloins)	–
47	Oxime-, methoxy-phenyl	Esters	–
48	2,2,4-Trimethyl-1,3-pentanediol diisobutyrate	Esters	–
49	Propanoic acid, 2-methyl-, 3-hydroxy-2,4,4-trimethylpentyl ester	fatty acid esters	–
50	Hexadecanoic acid, methyl ester	fatty acid esters	Shows cardioprotective effect against the ischemia/reperfusion (I/R) injury [70], antibacterial [71], counteracts cyclophosphamide cardiotoxicity [72]
51	Octadecanoic acid, 2-hydroxy-1,3-propanediyl ester	fatty acid esters	–
52	Ethane-1,1-diol dibutanoate	fatty acid esters	–
53	Benzoic acid, 2-ethylhexyl ester	benzoic acid esters	–
54	2-Octyl benzoate	benzoic acid esters	–
55	Benzoic acid 2-methylpentyl ester	benzoic acid esters	–
56	Benzoic acid, heptyl ester	benzoic acid esters	–
57	Benzoic acid, tridecyl ester	benzoic acid esters	–
58	Benzoic acid, undecyl ester	benzoic acid esters	–
59	1,2-Benzenedicarboxylic acid, bis(2-methylpropyl) ester	phthalate esters	–
60	Diibutyl phthalate	phthalate esters	–
61	Formic acid, octyl ester	fatty alcohol esters	–
62	1-Propen-2-ol, acetate	fatty alcohol esters	–
63	Oxirane, (methoxymethyl)-	heterocyclic ethers	–
64	(2-Aziridinylethyl)amine	amines	–
65	Cetene	alkenes	–
66	Benzoic acid	benzenoids	Antibacterial and antifungal [73]
67	Phenol	phenols	Disinfectant [74]
68	3(2H)-Thiophenone, dihydro-2-methyl-	tetrahydrothiophenes	–
69	1,4-Benzenediol, 2,6-bis(1,1-dimethylethyl)-	quinones	–

**Table 11:** Identification of the bacterial species based on the sequence similarities.

No.	Isolates	16S rRNA Amplified Region Length	Bacterial Species	NCBI Accession No.
1	BSS25	1420 bp	99% with *Bacillus thuringiensis* F3	MF135173
2	BSS21	1492 bp	99% with *Bacillus toyonensis* FORT 102	MG561363
3	BSS16	1452 bp	99% with *Bacillus acidiproducens* NiuFun	MF446886
4	BSS13	1474 bp	98% with *Bacillus cereus* WAB2133	MH169322
5	BSS12	1449 bp	99% with *Bacillus safensis* AS-08	JX849661

## Data Availability

The authors confirm that the data supporting the findings of this study are available within the article.
